# Effects of TLR Agonists on the Hypoxia-Regulated Transcription Factor HIF-1α and Dendritic Cell Maturation under Normoxic Conditions

**DOI:** 10.1371/journal.pone.0010983

**Published:** 2010-06-07

**Authors:** Rolf Spirig, Siamak Djafarzadeh, Tomas Regueira, Sidney G. Shaw, Christophe von Garnier, Jukka Takala, Stephan M. Jakob, Robert Rieben, Philipp M. Lepper

**Affiliations:** 1 Laboratory of Cardiovascular Research, Department of Clinical Research, University of Bern, Bern, Switzerland; 2 Laboratory of Intensive Care, Department of Clinical Research, University of Bern, Bern, Switzerland; 3 Laboratory of Vasoactive Peptides, Department of Clinical Research, University of Bern, Bern, Switzerland; 4 Department of Pneumology, Bern University Hospital, Bern, Switzerland; 5 Department of Intensive Care Medicine, Bern University Hospital, Bern, Switzerland; Fundação Oswaldo Cruz, Brazil

## Abstract

Dendritic cells (DC) are professional antigen presenting cells that represent an important link between innate and adaptive immunity. Danger signals such as toll-like receptor (TLR) agonists induce maturation of DC leading to a T-cell mediated adaptive immune response. In this study, we show that exogenous as well as endogenous inflammatory stimuli for TLR4 and TLR2 induce the expression of HIF-1α in human monocyte-derived DC under normoxic conditions. On the functional level, inhibition of HIF-1α using chetomin (CTM), YC-1 and digoxin lead to no consistent effect on MoDC maturation, or cytokine secretion despite having the common effect of blocking HIF-1α stabilization or activity through different mechanisms. Stabilization of HIF-1α protein by hypoxia or CoCl_2_ did not result in maturation of human DC. In addition, we could show that TLR stimulation resulted in an increase of HIF-1α controlled VEGF secretion. These results show that stimulation of human MoDC with exogenous as well as endogenous TLR agonists induces the expression of HIF-1α in a time-dependent manner. Hypoxia alone does not induce maturation of DC, but is able to augment maturation after TLR ligation. Current evidence suggests that different target genes may be affected by HIF-1α under normoxic conditions with physiological roles that differ from those induced by hypoxia.

## Introduction

DC are a unique leukocyte population of professional antigen presenting cells (APC) that play an important role in bridging innate and adaptive immunity [1]. They are crucial for inducing T-cell mediated immune responses, as seen in infection, allograft rejection, as well as the induction of peripheral tolerance [2], [3]. DC continuously scan their environment. For this purpose, they express pattern recognition receptors (PRR) including Toll-like receptors (TLR), nucleotide-binding oligomerization domain (NOD)-like receptors, C-type lectin receptors and others. Activating signals such as pathogen-derived molecules, e.g. LPS or lipoteichoic acid (LTA), induce maturation of the cells. Recent studies have shown that some of these PRR can also sense the presence of endogenous danger molecules, which are released in the context of tissue injury [4]. Ischemia/reperfusion (I/R) results in shedding and degradation of the glycosaminoglycans heparan sulfate (HS) [5]–[8] and hyaluronic acid (HA) [9], [10] from the endothelial cell surface. It has been shown that HS and HA induce maturation of DC via TLR4 *in vitro* [11], [12]. Furthermore, it has been demonstrated that TLR2 and TLR4 are crucially involved in I/R injury *in vivo* [13]–[17]. Additionally, maturation of DC is influenced by their microenvironment. Recent evidence suggests, that Toll-like receptor ligation can lead to stabilization of the transcription factor hypoxia-inducible factor 1α (HIF-1α) under normoxic conditions, most likely via NFκB [18], [19]. HIF-1α has been described as a key regulator of a broad range of cellular and systemic responses to hypoxic conditions. HIF-1α is also involved in myeloid cell-mediated inflammation [20]. Furthermore, HIF-1α is a master regulator of the bactericidal capacity of phagocytes [21]. Lipopolysaccharides (LPS) induce the expression of HIF-1α in murine macrophages [22] and dendritic cells (DC) [23]. Additionally, HIF-1α knock-out mice develop less clinical signs of sepsis [24]. These findings suggest the hypothesis that HIF-1α may also be an important mediator of inflammatory responses in the absence of hypoxia and that HIF-1α activation by TLR ligands under normoxic conditions might have functional consequences for human DC maturation and cytokine production. Thus, we investigated the effect of TLR2 and TLR4 activation by endogenous and exogenous ligands on the upregulation of the transcription factor HIF-1α, dendritic cell maturation and cytokine production in human monocyte-derived DC (MoDC) under normoxia. Results show that stimulation of human MoDC with exogenous as well as endogenous TLR agonists induces the expression of HIF-1α in a time-dependent manner. Interestingly, hypoxia alone does not induce maturation of DC, but is able to augment maturation after TLR ligation. Furthermore, we have investigated the effect of three previously described HIF-1α inhibitors (chetomin [CTM], digoxin and an inhibitor of both, NFκB and HIF-1α signaling, YC-1 on MoDC maturation and function.

## Results

### Upregulation of HIF-1α by exogenous and endogenous TLR4 agonists in human MoDC under normoxia

**Figure 1:**
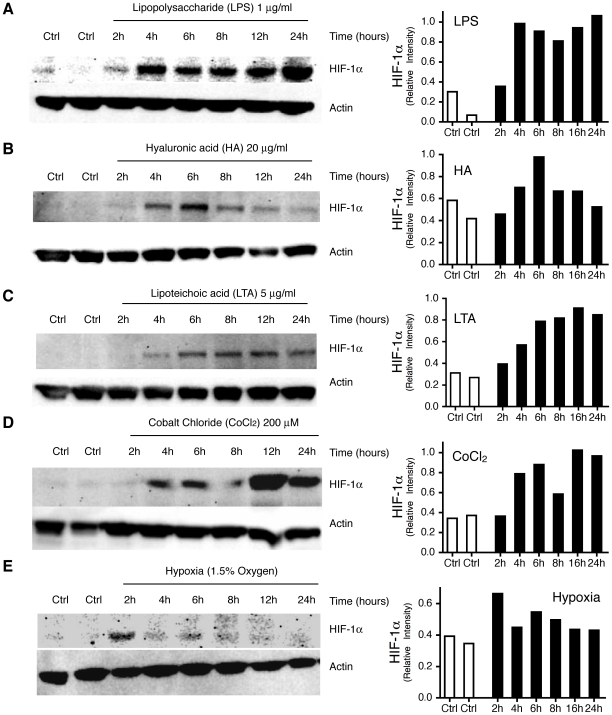
Time dependent stabilization of HIF-1α protein by TLR agonists, CoCl_2_ and Hypoxia. Monocytes isolated from buffy coat were cultured in presence of GM-CSF and IL-4 for 6 days. Cell lysates from MoDC that had been incubated with LPS (1 µg/ml, A), HA (20 µg/ml, B), LTA (5 µg/ml, C), CoCl_2_ (200 µM, D) or cultured under hypoxic conditions (1.5% oxygen, E) for the indicated periods were prepared. Equal amounts of protein were loaded and separated by SDS page, transferred onto membranes and probed for HIF-1α and actin. Relative Intensity Values are indicated on the right side of each blot. Protein Densiometry was quantified using Adobe Photoshop CS3. Values are representative for 3–4 experiments with cells from different donors.

**Figure 2:**
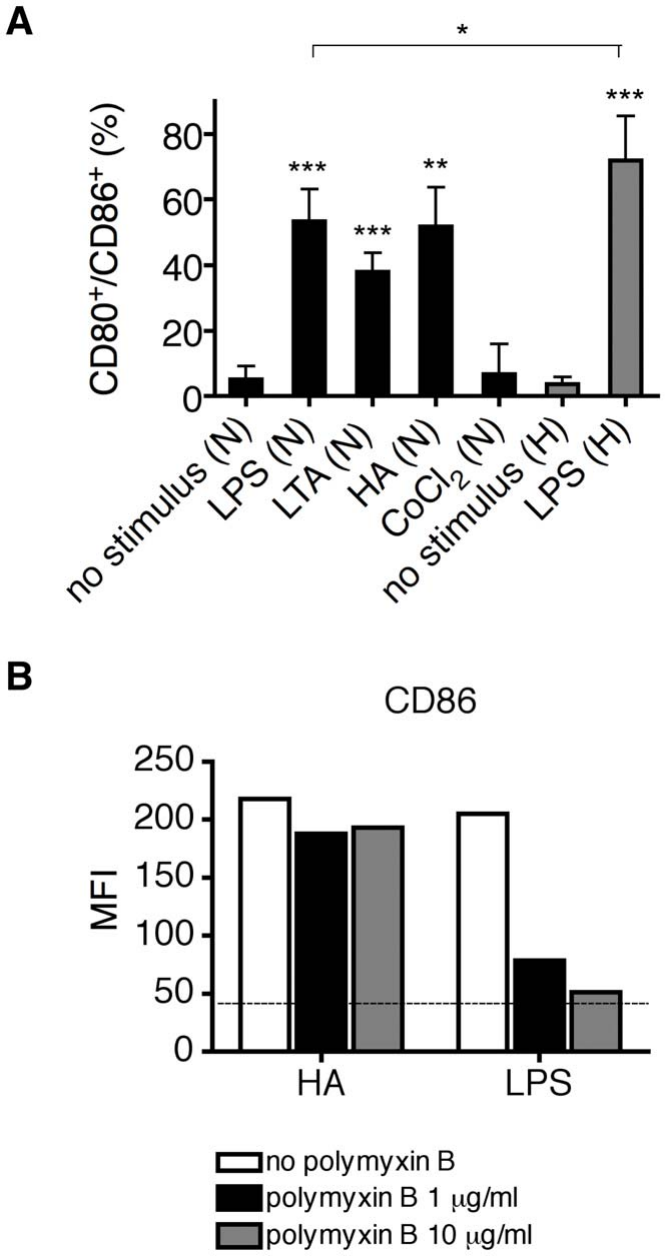
Evaluation of phenotypic maturation of MoDC. (A) Percentages of CD80^+^ CD86^+^ double-positive cells stimulated with LPS (1 µg/ml), HA (20 µg/ml), LTA (5 µg/ml), CoCl_2_ (200 µM) or cultured under hypoxia ± LPS are shown as mean values ± SD of 3–5 experiments with cells from different donors. N: Normoxia, H: Hypoxia. (B) Polymyxin B does not affect stimulatory activity of HA. MoDC were incubated with polymyxin B (1 or 10 µg/ml) and then stimulated with HA (20 µg/ml) or LPS (1 µg/ml) for 24 hours. Afterwards, cells were washed and analyzed for the expression of CD86 as a maturation marker. Y-axis shows the median fluorescence intensity (MFI). The dotted line indicates the MFI of non-stimulated MoDC. Data are representative of three independent experiments.

To investigate if HIF-1α is upregulated by TLR stimulation under normoxic conditions, MoDC were incubated with selective TLR ligands for the indicated time periods in a normal humidified cell incubator. We used LPS (1 µg/ml, Fig. 1A) and HA (20 µg/ml, Fig. 1B) as exogenous and endogenous TLR4 agonists, respectively. HIF-1α expression at the protein level was determined by Western blot analyses. As shown in Fig. 1A and B, both TLR4 agonists induced the expression of HIF-1α in a time-dependent manner. In parallel, cells were stained for the expression of the co-stimulatory molecules CD80 and CD86 as markers of cell maturation to confirm activation of the cells (Fig. 2A). To rule out contamination of HA with LPS, we incubated the cells with polymyxin B, an inhibitor of the biological activities of endotoxin. As shown in Fig. 2B, polymyxin B did not affect maturation of MoDC induced by HA whereas the effect of LPS was abolished.

### HIF-1α expression is induced by TLR2

To investigate, if stabilization of HIF-1α protein is restricted to TLR4 signaling, we tested the effect of the TLR2 agonist LTA on the expression of HIF-1α. As shown in Fig. 1C, LTA also induced the stabilization of HIF-1α protein in a time dependent manner under normoxic conditions.

### Hypoxia and stabilization of HIF-1α by the agonist CoCl_2_ does not induce maturation of MoDC

We further analyzed, if stabilization of HIF-1α protein by CoCl_2_ alone, without stimulation of TLR, leads to an activation of MoDC. As shown in Fig. 1D, treatment with CoCl_2_ led to the stabilization of HIF-1α but did not lead to a significant increase of the expression of the co-stimulatory molecules CD80 and CD86 (Fig. 2A). In addition we investigated the effect of hypoxic conditions (1.5% oxygen) on the maturation of MoDC. Hypoxia alone did not lead to phenotypic maturation of human MoDC (Fig. 2A), whereas the cells still matured if they were incubated together with LPS (Fig. 2A). MoDC stimulated with LPS under hypoxic conditions showed a synergistic response with a significant increase of double positive CD80^+^/CD86^+^ cells (Fig. 2A) compared to LPS stimulation under normoxia.

### Analysis of commonly used inhibitors of HIF-1α/NFκB (CTM, digoxin and YC-1) on phenotypic maturation of human MoDC

To further characterize the potential involvement of the transcription factor HIF-1α and NFκB in the maturation process of human MoDC, we used CTM (Chetomin), YC-1 and digoxin to attenuate HIF-1α and/or NFκB responses. CTM has been shown to block the interaction of HIF1α with transcriptional co-activators p300 and cAMP response element binding protein (CREB), thereby attenuating hypoxia-inducible transcription [25]. CTM, however, did not prevent the increase in NFκB p65 subunit nuclear levels or NFκB transactivation activity in response to LPS-IFNγ challenge [19].

YC-1 in contrast has been reported to abolish constitutive nuclear translocation and activation of NFκB/p65 [26] an upstream regulator of HIF-1α. However, YC-1 might also act on the PI3K/Akt/mTOR pathway, which can also regulate HIF-1α expression at the translational step [27]. Digoxin is thought to block accumulation of HIF-1α protein [28].

**Figure 3:**
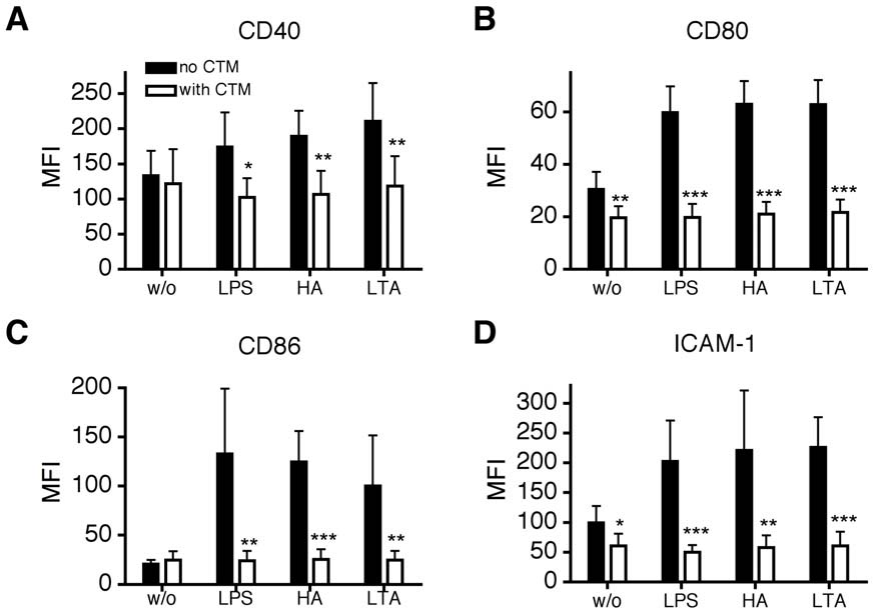
Effect of CTM on phenotypic maturation of MoDC. Monocytes isolated from buffy coat were cultured in the presence of GM-CSF and IL-4 for 6 days. Inhibition of HIF-1α expression by CTM inhibited maturation of MoDC in response to LPS. MoDC were incubated with CTM (200 nM) for 3 hours, afterwards cells were stimulated for 24 hours with LPS (1 µg/ml), HA (20 µg/ml) and LTA (5 µg/ml). Cells were washed and analyzed for the expression of CD40 (A), CD80 (B), CD86 (C) and ICAM-1 (D). Y-axis shows the median fluorescence intensity (MFI). Mean values ± SD of 6 experiments with cells from different donors are shown. *p<0.05; **p<0.01; ***p<0.001 vs. mature MoDC (unpaired Student's *t*-test).

**Figure 4:**
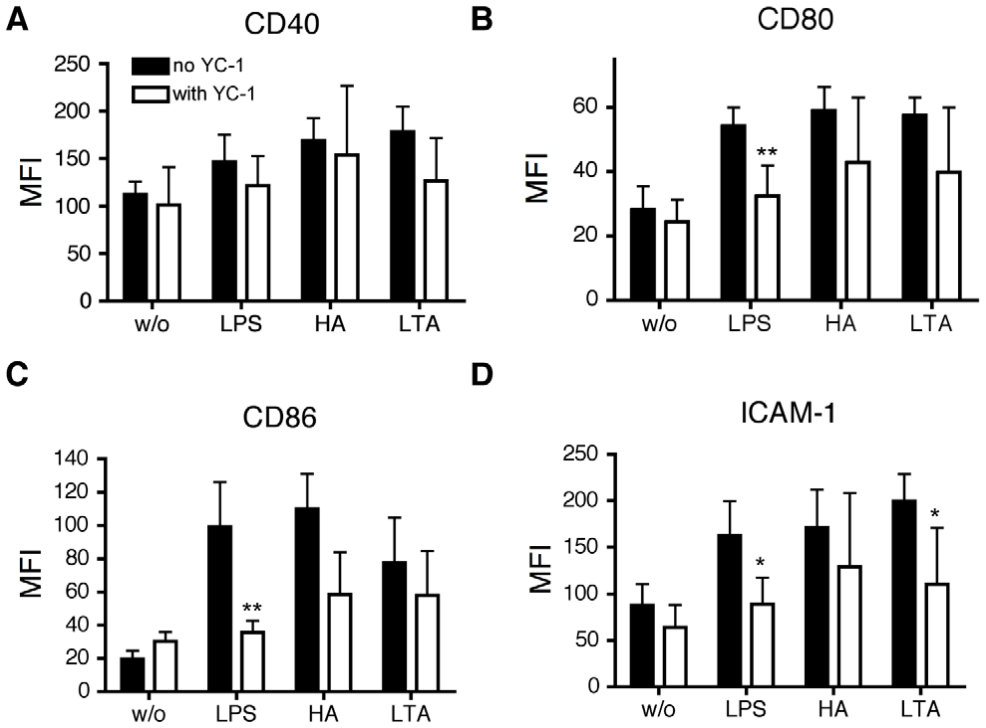
Effect of YC-1 on phenotypic maturation of MoDC. Monocytes isolated from buffy coat were cultured in the presence of GM-CSF and IL-4 for 6 days. MoDC were incubated with YC-1 (100 µM) for 5 min, afterwards cells were stimulated for 24 hours with LPS (1 µg/ml), HA µ20 (g/ml) and LTA (5 µg/ml). Cells were washed and analyzed for the expression of CD40 (A), CD80 (B), CD86 (C) and ICAM-1 (D). Y-axis shows the median fluorescence intensity (MFI). Mean values ± SD of 4 experiments with cells from different donors are shown. *p<0.05; **p<0.01 vs. mature MoDC (unpaired Student's t-test).

**Figure 5:**
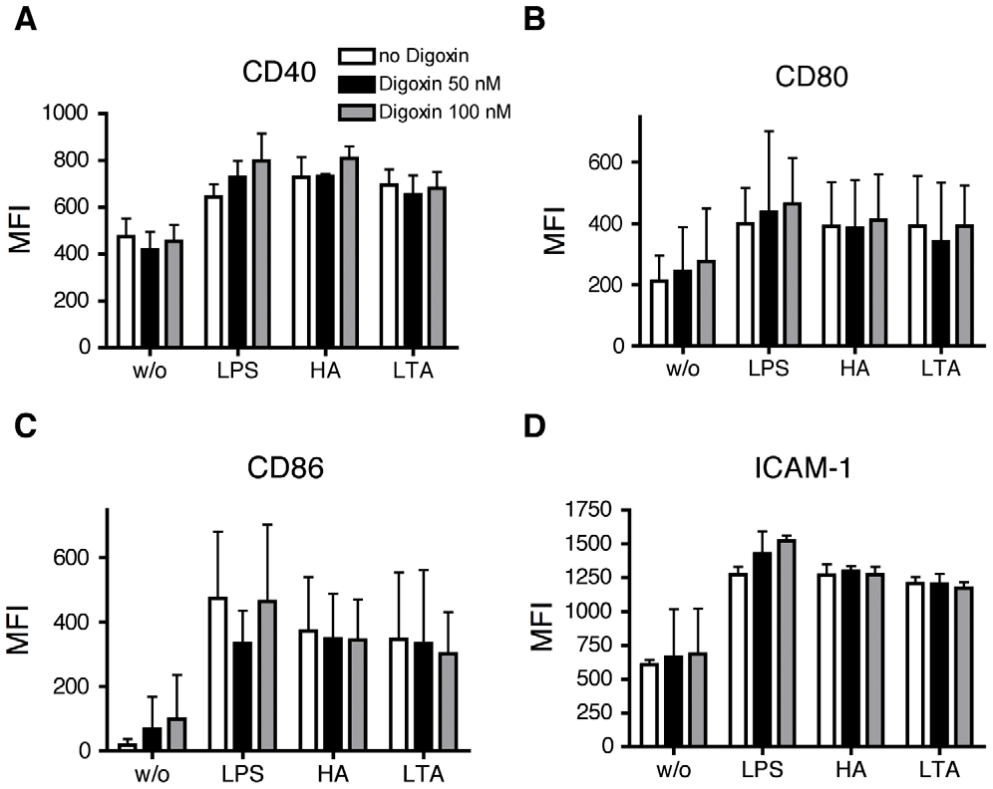
Effect of Digoxin (50 nM or 100 nM) on phenotypic maturation of MoDC. Monocytes isolated from buffy coat were cultured in the presence of GM-CSF and IL-4 for 6 days. MoDC were incubated with Digoxin for one hour, afterwards cells were stimulated for 24 hours with LPS (1 µg/ml), HA (20 µg/ml) and LTA (5 µg/ml). Cells were washed and analyzed for the expression of CD40 (A), CD80 (B), CD86 (C) and ICAM-1 (D). Y-axis shows the median fluorescence intensity (MFI). Mean values ± SD of 3 experiments with cells from different donors are shown.

MoDC were pretreated with CTM for 3 hours and then stimulated with LPS, LTA or HA for 24 hours. Afterwards, cells were analyzed for expression of CD40, CD80, CD86 and ICAM-1 (CD54) by flow cytometry. CTM consistently inhibited the up-regulation of all markers compared to the positive controls in response to all TLR ligands tested (Fig. 3A-D). In contrast, pretreatment of MoDC with YC-1 for 5 minutes before exposure to LPS, LTA or HA for 24 hours significantly inhibited only the up-regulation of CD80, CD86 and ICAM-1 primarily in response to LPS. No significant inhibitory response of YC-1 was observed for HA or LTA except for ICAM-1 induction by LTA (Fig. 4A-D). In a second series of experiments digoxin was used as an additional inhibitor of HIF-1α in an attempt to confirm the above observations. MoDC were pretreated with Digoxin (50 nM, 100nM) for one hour then stimulated with LPS, LTA or HA for 24 hours. As before, cells were analyzed for expression of CD40, CD80, CD86 and ICAM-1 by flow cytometry. Under these conditions, there was no significant effect of digoxin on the expression of any of the maturation markers analysed. (Figure 5A-D).

**Figure 6:**
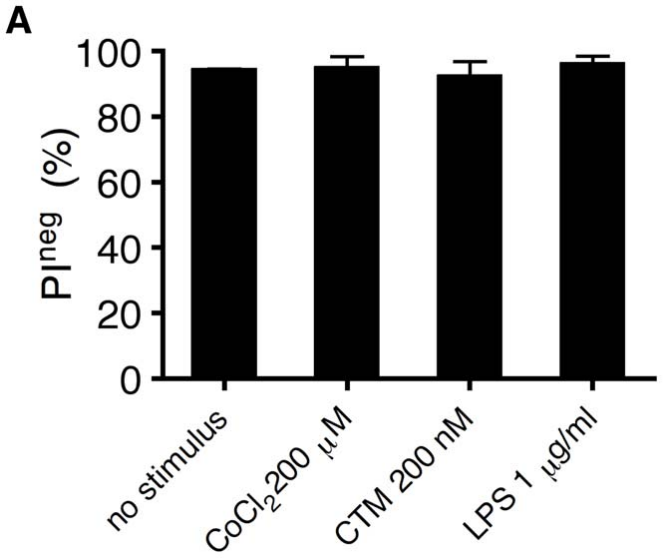
MoDC viability in response to CoCl_2_, CTM and LPS. Cells were treated with CoCl_2_, CTM and LPS for 24 hours. Thereafter cells were harvested and stained with PI (5 µg/ml). Unstained viable cells (PI^neg^) were enumerated by flow cytometry. Mean values ± SD of 3 experiments with cells from different donors are shown.

In all studies, exposure of MoDC to the used concentrations of CTM, CoCl_2_, LPS or YC-1 for 24hr did not affect viability of the cells as determined by flow cytometry (Figure 6). As a general rule any dead cells were always excluded from flow cytometric analysis by PI staining and there was no indication of synergistic loss of cell viability in co-incubation studies.

### Modification of VEGF secretion by MoDC by CTM, YC-1 and digoxin

**Figure 7:**
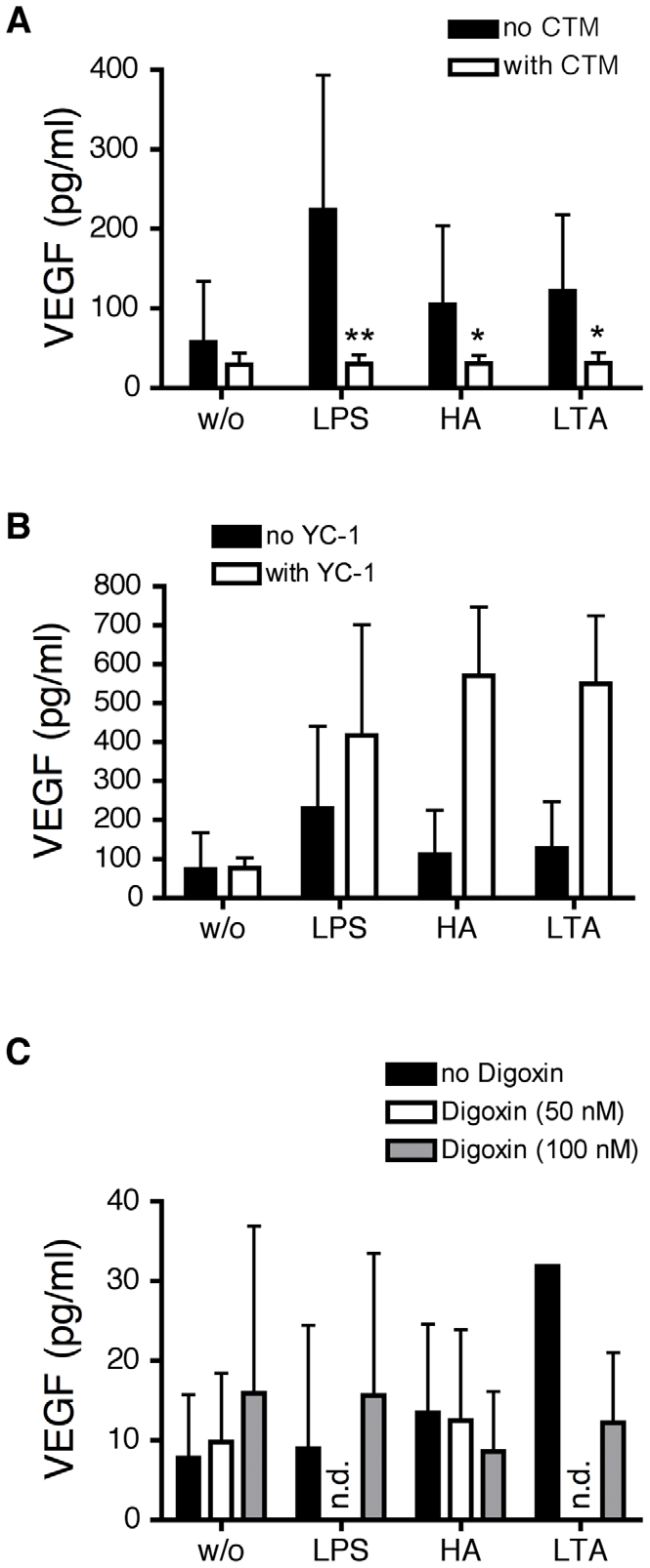
Effect of HIF-1α inhibition by CTM, YC-1 and Digoxin on VEGF production of MoDC. MoDC were incubated with CTM (200 nM) for 3 hours, YC-1 (100 µM) for 5 minutes and Digoxin (50 nM or 100 nM) for one hour, then cells were stimulated with LPS (1 µg/ml), HA (20 µg/ml) and LTA (5 µg/ml). After 24 hours cells were harvested and the supernatant assayed for VEGF with a Luminex multiplex array system. w/o: without stimulus. Mean values ± standard deviation of 6 experiments (4 for YC-1 and 3 for Digoxin) with cells from different donors are shown. *p<0.05; **p<0.01 vs. mature MoDC (unpaired Student's *t*-test).

Under hypoxic conditions, the *vegf*-gene is known to be at least partly under control of the transcription factor HIF-1α. To analyze if a similar dependency could be observed in response to TLR agonists under normoxic conditions, VEGF stimulation by selective TLR ligands was measured in the supernatants of MoDC. As shown in Fig. 7A, treatment with CTM significantly inhibited the TLR-induced production of VEGF whereas no inhibition was observed in the presence of YC-1 or digoxin. In fact, responses to HA and LTA in the presence of YC-1 were enhanced (Fig. 7B and C). As expected hypoxia-induced secretion of VEGF by MoDC was increased by incubation for 24 hours under hypoxic conditions (data not shown).

### Digoxin inhibits LPS-, HA- and LTA-induced increases in HIF-1α but does not alter TLR4 expression

**Figure 8:**
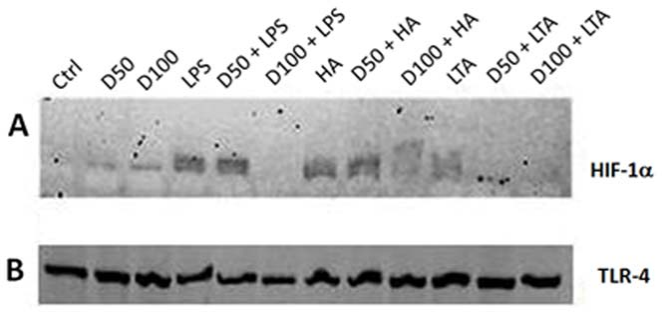
Digoxin inhibits LPS-, HA- and LTA-induced upregulation of HIF-1α (A) and does not modify TLR-4 expression (B). Cell lysates from MoDC that were incubated with digoxin (50 nM [D50] or 100 nM [D100]) and then stimulated with LPS (1 µg/ml), HA (20 µg/ml) or LTA (20 µg/ml) for 24 hours. Equal amounts of protein were loaded and separated by SDS page, transferred onto membranes and probed for HIF-1α and TLR-4.

Given the lack of effect of digoxin on expression of DC maturation markers or VEGF production in response to TLR ligands we determined the effect of digoxin on cellular HIF-1α protein levels. Cells were exposed to various concentrations of digoxin and thereafter stimulated with TLR ligands. As shown in Fig. 8A, pretreatment of cells with 50 and 100 nM digoxin dose dependently inhibited TLR agonist stimulated cellular HIF-1α protein increases whereas TLR4 levels were unaffected (Fig. 8B).

### Treatment of MoDC with CTM, YC-1 or digoxin differentially modulates secretion of proinflammatory cytokines and IL-10

The effect of HIF-1α/NFκB inhibitors on the secretion of cytokines by MoDC was evaluated using a Luminex multiplex array system. Supernatants of LPS, HA and LTA stimulated cells were assayed for IL-1β, IL-6, IL-10 and TNF-α (for digoxin IL-8 was also analyzed).

**Figure 9:**
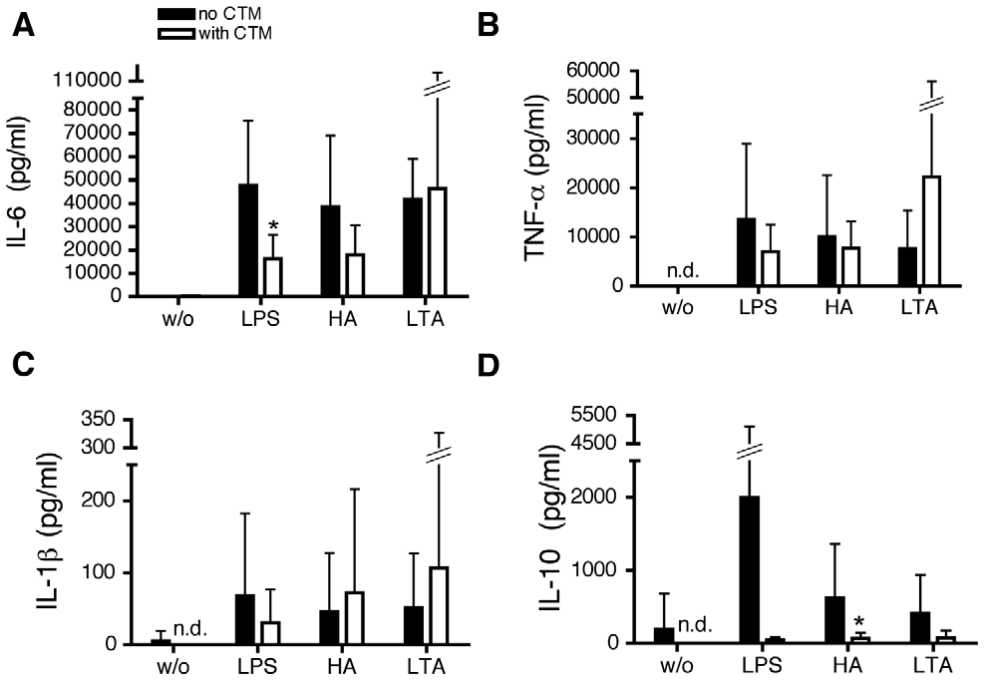
Modulation of proinflammatory cytokine secretion and IL-10 by inhibition of HIF-1α by CTM. (A), (B), (C) and (D), MoDC were stimulated with LPS (1 µg/ml), HA (20 µg/ml) and LTA (5 µg/ml). After 24 hours of incubation, cells were harvested and the supernatants examined for the amounts of IL-6 (A), TNF-α (B), IL-1β (C) and IL-10 (D) with a Luminex multiplex array system. w/o: without stimulus. Mean values ± SD of 6 experiments with cells from different donors are shown. *p<0.05 vs. mature MoDC (unpaired Student's *t*-test).

**Figure 10:**
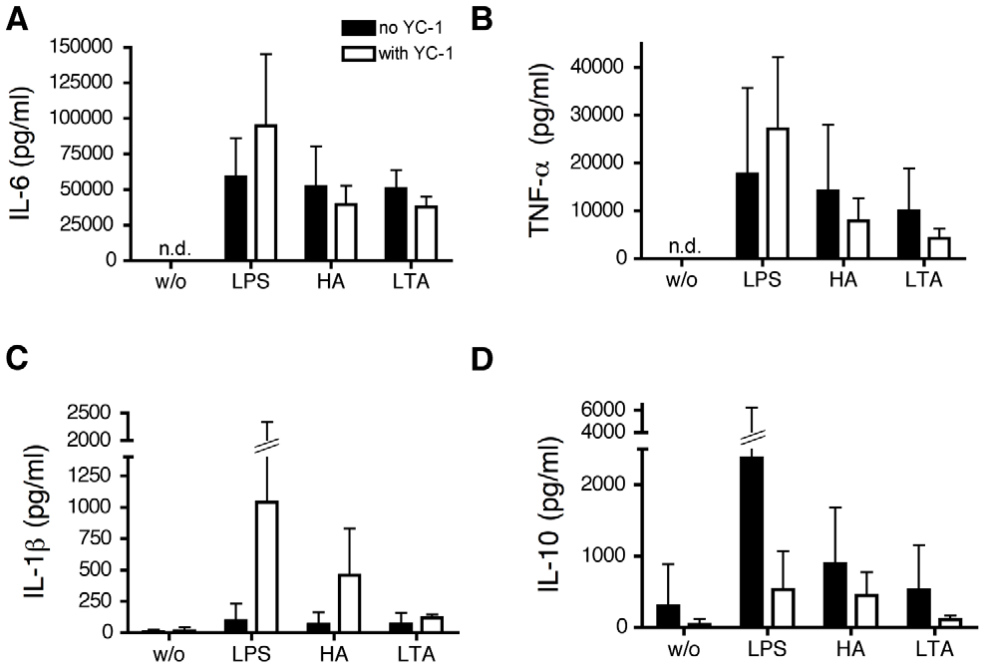
Modulation of proinflammatory cytokine secretion and IL-10 by YC-1. (A), (B), (C) and (D), MoDC were stimulated with LPS (1 µg/ml), HA (20 µg/ml) and LTA (5 µg/ml). After 24 hours of incubation, cells were harvested and the supernatants examined for the amounts of IL-6 (A), TNF-α (B), IL-1β (C) and IL-10 (D) with a Luminex multiplex array system. w/o: without stimulus. Mean values ± SD of 4 experiments with cells from different donors are shown.

**Figure 11:**
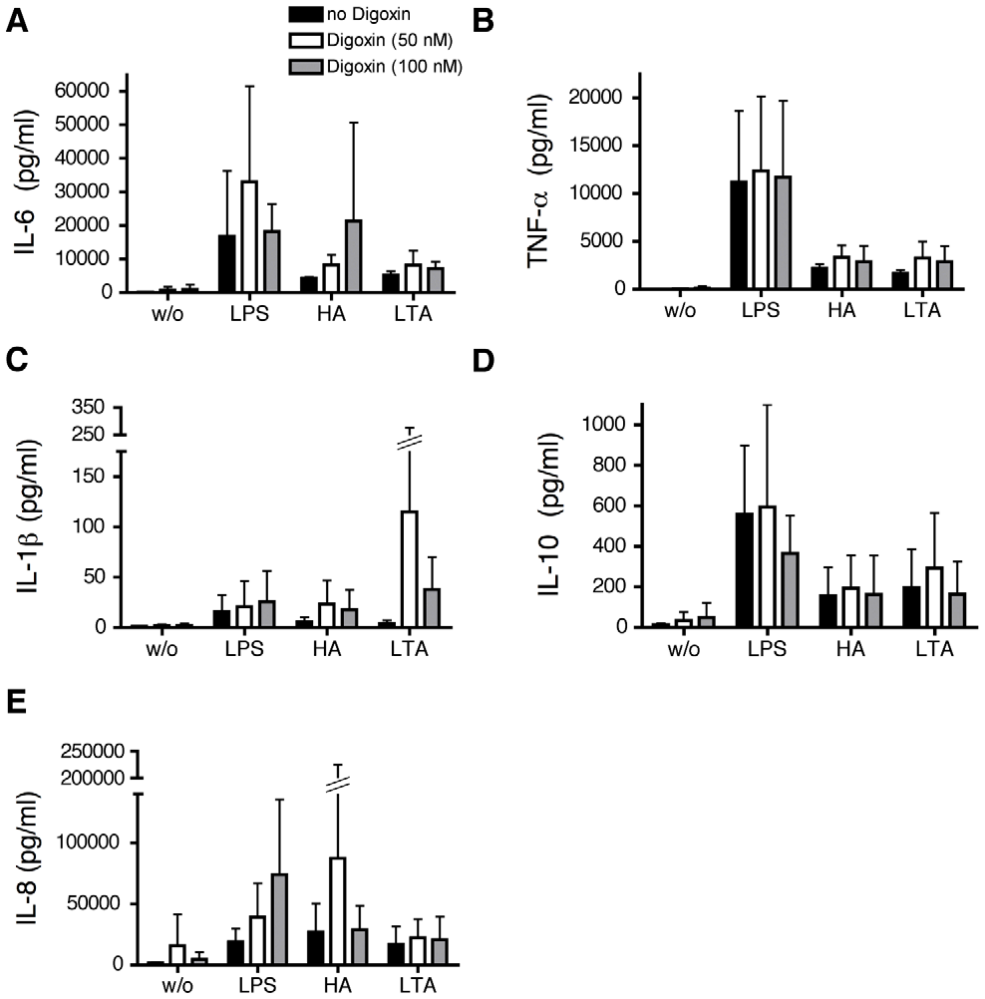
Modulation of the secretion of proinflammatory cytokines as well as IL-10 by inhibition of HIF-1α by digoxin. (A), (B), (C) and (D), MoDC were stimulated with LPS (1 µg/ml), HA (20 µg/ml) and LTA (5 µg/ml). After 24 hours of incubation, cells were harvested and the supernatants examined for the amounts of IL-6 (A), TNF-α (B), IL-1β (C), IL-10 (D) and IL-8 (E) with a Luminex multiplex array system. w/o: without stimulus. Mean values ± SD of 3 experiments with cells from different donors are shown. *p<0.05 vs. mature MoDC (unpaired Student's t-test).

Stimulation of MoDC with LPS resulted in a marked increase in the proinflammatory cytokine IL-6. which was inhibited approx. 75% by CTM treatment after 24 hours of stimulation. The trend towards a similar attenuated response to HA did not reach statistical significance (Fig. 9A). None of the TLR agonists significantly affected TNF-α or IL-1β production (Fig 9B and C) but treatment with CTM reduced IL-10 secretion in response to stimulation with the endogenous TLR4 ligands LPS and HA (Fig. 9D). In contrast no significant inhibitory effects of YC-1 or digoxin were observed for any of the cytokines analyzed (Fig. 10, 11).

### Effect of LPS stimulation on mitochondrial respiration of MoDC

**Figure 12:**
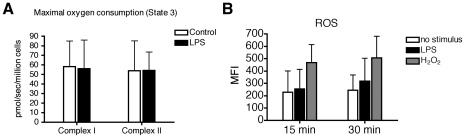
Influence of LPS stimulation on mitochondrial respiration and the generation of ROS by MoDC. (A) MoDC were stimulated under normoxic conditions for 4 hours with LPS (1 µg/ml). Mitochondrial Respiration was evaluated using a high resolution respirometry system. Mean values ± SD from 10 donors are shown as column graphs. (B) Prior to TLR stimulation, cells were incubated with 0.4 mM DCFH-DA for 15 minutes. Afterwards cells were washed and treated with LPS (1 µg/ml) or H_2_O_2_ (2 mM) for 15 and 30 minutes and fluorescence was evaluated by using flow cytometry. Y-axis shows the mean fluorescence intensity (MFI). Mean values ± SD from 3 donors are shown as column graphs.

As maturation is an energy dependent process, we analyzed the functional effect of TLR receptor stimulation on energy metabolism, and measured the mitochondrial respiration of MoDC, which leads to generation of ATP. DC were incubated with 1 µg/ml of LPS under normoxic conditions. After 4 hours of incubation no significant changes in maximal oxygen consumption (state 3) was observed (Fig. 12A)

### Influence of LPS on the generation of ROS in human MoDC

Since LPS can induce generation of ROS in human primary MoDC, which in turn might play a role in the maturation process we analyzed the generation of ROS using flow cytometry. We could not observe an increase in ROS after 15 and 30 minutes stimulation with LPS, whereas levels of ROS treated with the positive control hydrogen peroxide were increased (Fig. 12B).

## Discussion

The present study addressed the question of whether HIF-1α could be induced by different TLR agonists under normoxia and if stabilization is responsible for the subsequent MoDC maturation and TLR ligand-induced cytokine production under these conditions. The results demonstrate that under normoxic conditions, endogenous and exogenous TLR2- and TLR4-ligands induce maturation of human MoDC, which is associated with cell surface expression of CD40, CD80, CD86 and ICAM-1 in a time dependent manner together with secretion of VEGF and proinflammatory cytokines, and stabilization of the transcription factor HIF-1α.

Recent evidence suggests that endogenous TLR agonists play an important role in human disease states such as I/R injury which occurs in solid organ transplantation, myocardial infarction, autoimmunity or trauma. HA, a non-sulfated glycosaminoglycan, is a major constituent of the extracellular matrix (ECM). During tissue injury or inflammation, HA is degraded and shed from the ECM and cell surface and serves as an endogenous danger signal [9], [29]. Subsequent DC maturation and activation, with induction of proinflammatory cytokines, then contributes to a prolongation and exacerbation of tissue damage. Inflammatory responses of mouse macrophages to endogenous and exogenous TLR2 and TLR4 ligands [30] have previously been reported [31]. A potential role, however, or involvement of HIF-1α in these processes has not been considered. Untill now, HIF-1α protein stabilization under normoxic conditions by TLR ligands has been shown only for stimulation with LPS in murine bone-marrow derived DC, [23] and several studies have highlighted distinct differences between mouse and human immunology [32]. Hence the relevance of these earlier observations for human disease has largely remained unknown. In the present experiments, LTA from the gram-positive cocci *S. aureus*, a TLR2 ligand [33], induced DC maturatuion and HIF-1α protein stabilization, comparable to the stimulation induced by LPS from the gram-negative rod *E. coli*. Similar effects were induced by the endogenous TLR4 ligand HA although some differences were observed in the time course. A possible explanation may relate to the known differences in requirement of HA and LPS for distinct accessory molecules leading to alternate downstream signaling pathway activation (Taylor 2007).

In contrast, neither hypoxia nor CoCl_2_ induced HIF-1α accumulation lead to phenotypic maturation of MoDC examined after 24 hours suggesting that changes in HIF-1α per se do not necessarily lead to maturation. At first sight this may suggest methodological differences as an underlying factor. However, at the dose used, CoCl_2_ has clearly been shown to be an inducer of HIF-1α activation [34]–[36]. Furthermore, different ranges of oxygen levels have been considered as hypoxic conditions, starting from almost anoxic conditions of 0.1% up to 2% O_2,_ oxygen levels in the body vary from around 16% in the pulmonary alveoli down to approximately 6% in other tissues [37]. Although there is no current consensus regarding a critical threshold level we used the commonly quoted value of 1.5% [38], [39]. While this level of hypoxia did not directly lead to MoDC maturation we did observe a synergistic effect on LPS induced phenotypic maturation of MoDC in the presence of hypoxia. Consequently, hypoxia may augment an inflammatory response elicited in the presence of a danger signal associated with infection or ischemia. In contrast, hypoxia without pro-inflammatory stimuli does not induce activation, which may serve as a protection mechanism of the host preventing development of an autoimmune response. These data are consistent with the finding of Jantsch *et al.* in murine DC for hypoxia [23]. In contrast, a recent study has shown that human MoDC differentiated from monocytes under permanent hypoxic (1% O_2_) conditions exhibit a reduced up-regulation of CD40, CD80, CD83 and CD86 in response to LPS, whereas the secretion of TNF-α, CCL22 and IL-1β was increased [40]. Another study demonstrated, that MoDC generated under hypoxic conditions (1% O_2_) change their chemokine releasing profile and exhibit a reduced Ag-uptake capacity [41]. These differences may relate to the fact that hypoxia during the differentiation process of monocytes into DC has additional effects on cells that differ from hypoxic exposure of already fully differentiated DC.

**Figure 13:**
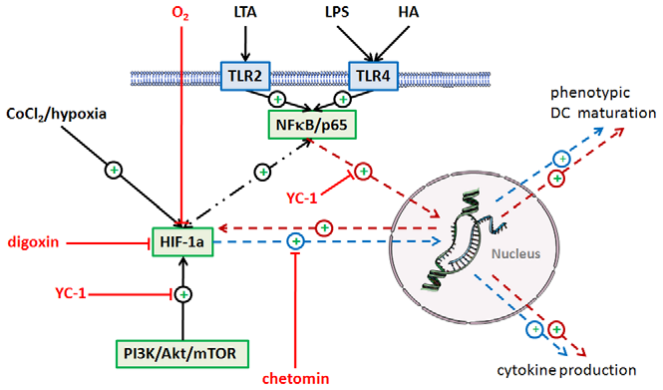
Summary of the effects of NFκB and HIF-1α on DC maturation. Ligation of TLRs leads to NFκB activation and subsequent upregulation of HIF-1α under normoxic conditions. HIF-1α contributes to NFκB driven phenotypic DC maturation and cytokine production. The effects were investigated using an inhibitor of NFκB/p65 nuclear translocation (YC-1), inhibition of HIF-1α nuclear translocation (chetomin), and inhibition of HIF-1α protein accumulation (digoxin). CoCl2 and hypoxia served as controls.

In view of the complex interplay between these factors we subsequently evaluated a potential role of HIF-1α stabilization in the processes of MoDC maturation and cytokine production using three inhibitors of HIF-1α and/or NFκB, namely CTM [25], YC-1 [27] and digoxin [28] (see Fig. 13).

Taken together the data showed that there is no consistent effect of the three inhibitors on MoDC maturation, or cytokine secretion despite having the common effect of blocking HIF-1α stabilization or activity through different mechanisms. Thus the data of YC-1 and digoxin did not support the original hypothesis that HIF-1α activation by TLR ligands under normoxic conditions may have functional consequences for human MoDC maturation, VEGF and cytokine production. These events appear to occur independently of the observed changes in HIF-1α stabilization. This would suggest that other, as yet unknown effects of these inhibitors are responsible for differential responses to the individual inhibitors in relation to cell maturation (cell surface marker expression, or VEGF and cytokine production). This is reinforced by the observation that digoxin was without effect on any of the parameters measured despite preventing TLR induced stabilization of HIF-1α in response to LPS, LTA and HA.

Similarly variable responses of the antagonists were seen for VEGF production by TLR ligands where only CTM showed any inhibition and responses to HA and LTA in the presence of YC-1 were even enhanced.

In this regard, non-HIF-1α dependent stimuli including insulin-like growth factor-1 (IGF-1), also stimulate VEGF secretion which is phosphatidyl-inositol 3-kinase (PI3K)/Akt/mTOR (mammalian target of rapamycin)-dependent. Other regulators include angiotensin, nitric oxide and guanyl cyclases [42], [43]. Interestingly YC-1 was originally characterized as a cGMP inducer because it stimulated soluble guanylyl cyclase activation in response to nitric oxide or carbon monoxide [44] in some cells, only low concentrations of YC-1 (1–20 µmol/L) are required for anti-HIF-1α activity, whereas cGMP elevation requires higher concentrations (>50 µmol/L; [44] as used in the present study. Whether and to what extent this may play a role in the differential responses of MoDC to YC-1 in the presence of different TLR ligands, particularly in relation to the observed stimulatory effects on VEGF remain to be determined.

In general, different pharmacokinetic properties (accessibility, excretion, or metabolism) and characters of target molecules (expression, activation, or isoforms) are considered major factors determining drug sensitivity. However, these aspects in relation to CTM, YC-1 and digoxin have not been fully determined to date; thus, the underlying basis for the varying sensitivity and responsiveness of the maturation process, cytokine production and VEGF secretion to the different inhibitors remains to be investigated.

This begs the question of whether HIF-1α stabilization under normoxic conditions has other physiological consequences and whether the role of downstream events that may be activated are the same as in hypoxia. Current evidence suggests that different target genes may be affected by HIF-1α under normoxic vs hypoxic conditions with physiological roles that differ from those induced by ischemia. Further investigation of these aspects of HIF-1α action, however, lie outside the original scope and aims of the present study.

## Materials and Methods

### Generation and stimulation of human monocyte-derived DC (MoDC)

Human peripheral blood mononuclear cells (PBMC) were isolated from buffy coats obtained from healthy blood donors (Regional Red Cross Blood Donation Center, Bern, Switzerland) by density gradient centrifugation over Ficoll-Paque (Amersham, Uppsala, Sweden). Monocytes were isolated from PBMC as previously described [45]–[47] or by CD14 positive selection by MACS (Miltenyi Biotec GmbH, Bergisch Gladbach, Germany) according the manufacturer's protocol (for experiments with digoxin). Purity of isolated monocytes was characterized by high expression of CD14 (>96% positive cells) and absent expression of CD1a and DC-SIGN. Monocytes were incubated for 6 days in RPMI 1640 medium (Invitrogen Life Technologies, Basel, Switzerland) containing 10% fetal calf serum ((FCS, Amimed / BioConcept, Allschwil, Switzerland), 1% [2 mM] L-Glutamine (Invitrogen), 1% [100 U/ml] Penicillin/Streptomycin (Invitrogen), 10 ng/ml GM-CSF (R&D Systems Europe Ltd, Abingdon, Oxon, UK), and 10 ng/ml IL-4 (R&D) to generate MoDC as described initially by Sallusto and Lanzavecchia [48]. Immature MoDC were characterized by absent expression of CD14 and high expression of CD1a, HLA-DR, DC-SIGN and phagocytic activity. Maturation of these cells leads to a massive up-regulation of CD40, CD80, CD83, CD86, ICAM-1, CCR7 and HLA-DR. Mature MoDC exhibit a very potent T cell proliferation capacity and reduced Ag-uptake as previously described [46], [47], [49]. Cells were kept at 37°C in a 5% CO_2_ humidified atmosphere. On day 3, the culture medium was replaced with fresh medium. For induction of maturation 1 µg/ml LPS (Sigma Aldrich, Buchs, Switzerland), 5 µg/ml LTA or 20 µg/ml HA (Sigma Aldrich, Buchs, Switzerland) were added to the MoDC cultures for the indicated periods.

### FACS analysis and cell viability

Cells were incubated with FITC- or PE-labeled monoclonal antibody (mAb) against CD80, CD86 (BD, Franklin Lakes, NJ, USA), isotype control IgG1 (BD), or unlabeled mAb against CD40, ICAM-1 (Diaclone, Besançon, France) followed by a FITC-labeled polyclonal goat anti-mouse IgG (Sigma).

For determination of viability, the cells were stained with 5 µg/ml of propidium iodide (PI; Invitrogen) and analyzed by flow cytometry. As positive control for PI staining, cells were treated with PBS containing 0.1% BSA and 0.1% saponin (Sigma Aldrich, Buchs, Switzerland). Measurements were performed with a BD FACScan flow cytometer and analyzed using the FlowJo software (Tree Star Inc., Ashland, OR, USA).

### Hypoxic conditions

Cells were incubated in a hypoxia chamber (1.5% oxygen, 37°C) for the indicated time-periods. In some experiments (as indicated), LPS (1 µg/ml) was given in the hypoxia-chamber one hour after putting the cells under hypoxic conditions.

### SDS-PAGE and Western blotting

DC were lysed in 60 mM Tris-HCl, 8.5% glycerol, and 2% SDS. Protein concentration was determined with the Quanti-IT assay kit and read with the Qubit fluorometer (Invitrogen). Equal amounts of protein (20 µg per lane) were loaded and separated by 4–12% SDS-PAGE. Gels were then transferred to nitrocellulose membranes with the iBlot dry blotting system (Invitrogen). Equal loading was verified by staining the extracted gel with SimplyBlue SafeStain (Invitrogen). Afterwards, the membranes were blocked for 30 min with incubation buffer (10 mM Tris-HCl pH 7.5, 100 mM NaCl and 0.1% w/v Tween 20) supplemented with 5% non-fat dry skim milk, and incubated overnight with the primary antibodies against HIF-1α (Novus Biologicals, Littleton, CO, USA; dilution 1∶2000) or primary antibodies against TLR-4 (Invitrogen, Basel, Switzerland; dilution 1∶1000) and actin (Sigma Aldrich, Buchs, Switzerland; dilution 1∶3000). Membranes were washed with incubation buffer and incubated for 1 hour with horseradish peroxidase-coupled goat polyclonal anti-rabbit IgG (dilution 1∶3000). Finally, the membranes were developed with a chemiluminescence detection kit (Pierce, Rockford, IL, USA). All Western blotting experiments were performed in triplicates. Protein Densiometry was quantified using Adobe Photoshop CS3.

### Cytokine assays

MoDC (10^6^ cells/ml) were treated with LPS, LTA or HA for 24 hours. Cell culture supernatants were analyzed using a Luminex multiplex suspension array system from Bio-Rad (Bio-Rad, Hercules, CA, USA) for IL-1β, IL-6, IL-8, IL-10, TNF-α and VEGF (all kits from BioSource, Invitrogen, Carlsbad, CA, USA) according the manufacturer's instructions.

### Inhibitors and agonists of HIF-1α and/or NFκB

Blocking of HIF-1α was achieved using the commercially available inhibitors CTM (Chetomin, NSC 289491, Invitrogen), and digoxin (Sigma-Aldrich, Buchs, Switzerland). Chetomin is a natural metabolite produced by several species of the genus *Chaetomium*. To inhibit NFκB/p65 nuclear translocation, YC-1 (3-(5′-Hydroxymethyl-2′-furyl)-1-benzyl indazole; Calbiochem-Novabiochem, Zug, Switzerland) was used. Stabilization of HIF-1α protein in control experiments was achieved using CoCl_2_ (Sigma Aldrich, Buchs, Switzerland), which is a potent inhibitor of post-translational hydroxylation of proline residues in the oxygen-dependent degradation (ODD) domain of HIF-1α and thus inhibits HIF-1α degradation.

### High-resolution respirometry

DC were centrifuged for 5 min (350 g) and resuspended in respiration buffer (110 mM sucrose, 0.5 mM EGTA, 3.0 mM MgCl_2_, 80 mM KCl, 60 mM K-lactobionate, 10 mM KH_2_PO_4_, 20 mM taurine, 20 mM HEPES, 1.0 g/l BSA, pH 7.1) at a concentration of 3-5×10^6^ cells/ml. Cells were incubated with 1 µg/mL LPS for 4 h. Respiration rates were measured at 37°C in a high-resolution oxygraph (Oxygraph-2k, Oroboros Instruments, Innsbruck, Austria). For assessment of mitochondrial complex activity cells were first permeabilized with digitonin (8.1 µM) for 5 min. Afterwards, for complex I-dependent maximal respiration stimulation, substrates added were glutamate (10 mM) and malate (5 mM), followed by addition of adenosine diphosphate (ADP) (0.25 mM) causing a sudden burst of oxygen uptake as ADP is converted into ATP. After a stable signal was reached and marked, rotenone (0.5 µM) was added to inhibit complex I, and then complex II-dependent respiration was stimulated by adding succinate (10 mM), while complex III was inhibited by antimycin A (0.5 µM) (Sigma Aldrich, Buchs, Switzerland). Respiration rates were calculated and recorded using DatLab software for data acquisition and analysis; Oroboros Instruments, Innsbruck, Austria.

### Measurement of reactive oxygen species (ROS) generation by flow cytometry

MoDC (2×10^5^ cells) were plated in 96-well round bottom well plate. Cells were pretreated with 0.4 mM of to dichlorofluorescin diacetate (DCFH-DA [Sigma]) for 15 minutes. Afterwards, cells were washed and stimulated with LPS (1 µg/ml) or hydrogen peroxide (2 mM, Sigma) for 15 and 30 minutes and immediately analyzed by flow cytometry.

### Statistical analysis

Data are presented as mean ± standard deviation (SD) representing experiments with up to 10 different donors. Unpaired Students *t-tests* were performed for evaluation of significance. Cellular respiration was compared using the paired t-test. Differences were considered as statistically significant at p-values less than 0.05. Data were analyzed using GraphPad Prism software 4.0 (GraphPad, San Diego, CA).
